# Magnetic Properties of Novel Layered Disulfides CuCr0.99Ln0.01S2 (Ln = La…Lu)

**DOI:** 10.3390/ma14175101

**Published:** 2021-09-06

**Authors:** Evgeniy V. Korotaev, Mikhail M. Syrokvashin, Irina Yu. Filatova, Valentina V. Zvereva

**Affiliations:** Nikolaev Institute of Inorganic Chemistry, Siberian Branch of Russian Academy of Sciences, 630090 Novosibirsk, Russia; syrokvashin@niic.nsc.ru (M.M.S.); rare@niic.nsc.ru (I.Y.F.); zvereva@niic.nsc.ru (V.V.Z.)

**Keywords:** layered copper-chromium disulfide, lanthanides, magnetic properties, order-to-disorder transition, DSC

## Abstract

The comprehensive study of the lanthanide-doped solid solutions CuCr_0.99_Ln_0.01_S_2_ (Ln = La…Lu) magnetic properties was carried out using static magnetochemistry and differential scanning calorimetry techniques. It was shown that magnetic properties of CuCr_0.99_Ln_0.01_S_2_ are significantly affected by the magnetic properties of the lanthanide ion. The magnetic susceptibility and the effective magnetic moment were found to deviate from the Curie-Weiss law in the temperature 90 K below and 50 K above the order-disorder transition at 695 K. The observed behavior of the temperature dependence of the effective magnetic moment in the order-disorder transition temperature region was described as a result of copper atoms redistribution over different types of the crystallographic sites.

## 1. Introduction

The main trends of modern material science are the improvement of existing and the development of new functional materials. Layered chromium dichalcogenides CrX_2_-based (X = S, Se, Te) compounds can be considered as promising functional materials. The unstable dichalcogenide CrX_2_-layers can be stabilized by the intercalation of electropositive metal atoms between the adjacent dichalcogenide layers [1,2]. The functional properties of the layered CrX_2_-based materials could be modified by the cationic substitution of chromium atoms in the dichalcogenide layers or by simultaneous intercalation of metal atoms of different types between CrX_2_-layers. For instance, in CuCrS_2_, the unstable CrS_2_-layers are stabilized by copper atoms. This results in the layered structure of CuCrS_2_ that is formed by the alternating copper and dichalcogenide layers. The CuCrS_2_-based solid solutions CuCr_1-x_M_x_S_2_ are promising functional materials with thermoelectric, superionic, and magnetic properties [3,4,5,6,7,8,9,10]. Chromium atoms could be substituted with other 3*d*-metal atoms over a wide concentration range (x ≤ 0.40) without changes in the crystal structure of the initial matrix [6,11,12]. The cationic substitution of CuCrS_2_-matrix with heavier 3*d*- or 4*f*-metal atoms allows one to decrease the thermal conductivity due to the phonon scattering increase [8,13,14]. The reported thermoelectric properties of CuCr_1-x_M_x_S_2_ allow one to consider these compounds for the fabrication of high-performance thermoelectric generators (TEGs) [7,8]. The redistribution of copper atoms over the different sites in CuCr_1-x_M_x_S_2_-structure allows one to consider CuCrS_2_-based solid solutions as promising materials for solid-state power sources, ion-selective membranes, and chemical sensors [6,15,16]. The magnetic properties of CuCrS_2_-based solid solutions can be used for the fabrication of spin gates, filters, and magnetic memory devices [5,17].

The magnetic properties of the initial CuCrS_2_-matrix are related to the ferromagnetic ordering of the magnetic moments of chromium atoms in CrS_2_-layers and to the antiferromagnetic ordering of the adjacent layers. The antiferromagnetic ordering in CuCrS_2_ could be observed at temperatures below the Neel temperature (T_N_ ≈ 40 K). Above T_N_ temperature, CuCrS_2_-matrix and CuCr_1-x_M_x_S_2_ solid solutions become paramagnetic and their magnetic susceptibility follows the Curie-Weiss law [2,8,17,18,19,20,21]. As was mentioned above, the phonon scattering has a significant impact on the thermoelectric properties of the material. The phonon scattering enhancement due to the presence of the atoms bearing magnetic moment improves the thermoelectric properties [22]. The cationic substitution of chromium by atoms with different magnetic moment values should significantly affect the phonon scattering efficiency. The magnetic moment of Ln ions is significantly changed across the lanthanide series. Thus, the magnetic properties of the lanthanide-doped CuCr_1-x_Ln_x_S_2_ solid solutions are of special interest. As was previously reported, the optimal doping concentration for CuCrS_2_-based solid solutions is x = 0.01 [7,8]. An increase of x causes the Seebeck coefficient suppression due to the metal-insulator transition (MIT). Hence, here we report the study of magnetic properties of the lanthanide-doped CuCr_0.99_Ln_0.01_S_2_ (Ln = La…Lu) solid solutions.

CuCrS_2_-based materials exhibit a high-temperature order-disorder transition (ODT). The ODT affects the transport and thermoelectric properties in the high-temperature region (T > 670 K). As the temperature increases, the mobile metal atoms occupy sites that were unoccupied at room temperature. As a result, the electrical conductivity increases. The redistribution of the mobile metal atoms over crystallographic sites in CrS_2_-sublattice increases a structural disorder. The structural disorder reduces the lattice thermal conductivity resulting from the phonon scattering. Note that the ODT does not significantly affect the crystallographic structure of CuCrS_2_-matrix and has a reversible character. The magnetic susceptibility temperature dependence of CuCr_1-x_M_x_S_2_ follows the Curie-Weiss law in the temperature region above and below the ODT [2,19]:

(1) $$\chi \left(T\right)=\frac{C}{T-\theta }=\frac{N_{A}\mu_{B}^{2}}{3k\left(T-\theta \right)}\mu_{\mathrm{eff}}^{2}$$

where T is temperature, k is the Boltzmann constant, N_A_ is the Avogadro number, μ_B_ is the Bohr magneton, μ_eff_ is the effective magnetic moment, and θ is Weiss constant:

(2) $$\theta =\frac{2j\left(j+1\right)}{3k}\sum z_{i}J_{i}$$

where j is the total angular momentum quantum number, z_i_ is the magnetic coordination number, J_i_ is the exchange interaction between magnetic centers, and i is the magnetic center number.

However, it should be noted that reported data do not include a detailed study of the magnetic properties in the ODT temperature region. The typical temperature resolution in the corresponding region exceeds 50 degrees [2,8,19]. Nevertheless, the physical properties of CuCrS_2_-matrix dramatically change in the immediate vicinity of the ODT (about ±50 degrees) [3]. Thus, the detailed study of the magnetic properties of CuCr_0.99_Ln_0.01_S_2_ in the immediate vicinity of the ODT is of special interest and has not been reported yet.

The synthesis procedure significantly affects the magnetic properties of CuCrS_2_-matrix. The different experimental works report on the contradictory results concerning the effective magnetic moment (µ_eff_) and Weiss constant (θ) of CuCrS_2_. Thus, µ_eff_ and θ lie within the wide range of values 3.8 to 5.5 µ_B_ and −110 to −150 K, respectively. Furthermore, in case of the cation substituted solid solutions based on CuCrS_2_-matrix, the Weiss constant values are spread out even more [4,6,7,18,23]. For instance, some of the CuCrS_2_-based solid solutions were reported to have positive θ values [24]. This fact indicates the presence of a significant ferromagnetic contribution to the magnetic susceptibility value. It should be noted that the corresponding contribution could be due to the presence of specific ferromagnetic impurities [21]. Thus, in this study, the ferromagnetic contribution in the magnetic susceptibility value was taken into account.

Here, we report the study of the ODT and cationic substitution effect on the magnetic properties of lanthanide-doped CuCr_0.99_Ln_0.01_S_2_ (Ln = La … Lu) solid solutions using both differential scanning calorimetry (DSC) and static magnetochemistry techniques.

## 2. Experimental

**Figure 1:**
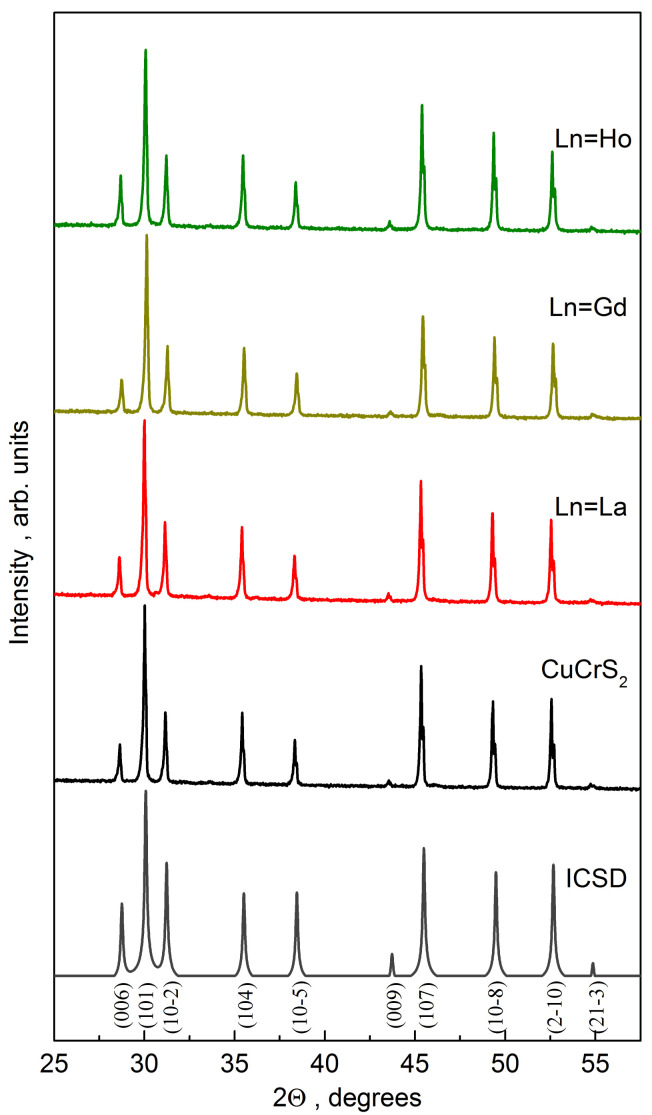
Powder diffraction patterns of CuCrS_2_-matrix and CuCr_0.99_Ln_0.01_S_2_ solid solutions (Ln = La, Gd, Ho).

The powder samples studied were synthesized from the initial metal oxides using a previously reported procedure [3,8]. The average grain size was 25 µm. The X-ray powder diffraction (XRD) experiment was carried out using non-monochromatic CuKα-radiation on a Shimadzu XRD 7000S diffractometer (Shimadzu Corporation, Kyoto, Japan). The XRD patterns were found quite similar. Figure 1 shows typical XRD patterns of the initial matrix and some solid solutions CuCr_0.99_Ln_0.01_S_2_. The XRD patterns of all CuCr_0.99_Ln_0.01_S_2_ (Ln = La…Lu) synthesized samples are shown in Supplementary Figure S1. The observed diffraction peaks on the XRD pattern indicate that the synthesized CuCrS_2_ sample has a rhombohedral structure with an R3m space group. The absence of the additional diffraction peaks allows one to conclude that synthesized samples are single-phase. The position and diffraction peaks intensity are in good agreement with the XRD data of the Inorganic Crystal Structure Database (database code 100594, denoted as “ICSD” in Figure 1) [25]. The calculated unit cell parameters *a* = 3.48(3) and *c* = 18.71(6) Å correlate well with the previously reported and reference data [7,25,26]. The lattice parameters of CuCr_0.99_Ln_0.01_S_2_ are close to those for CuCrS_2_-matrix and lies within the range of 3.47–3.48 and 18.67–18.71 Å for *a* and *c*, respectively. However, the unit cell volume slightly decreases due to the lanthanide contraction with an increase of the Ln atomic number (Supplementary Table S1 in Supplementary Materials). Thus, one can conclude that cationic substitution by lanthanides does not significantly affect the initial matrix crystal structure.

The elemental composition of the samples studied was controlled by X-ray fluorescence analysis using a Bruker MISTRAL M1 (Bruker Corporation, Billerica, MA, USA). The composition of the investigated solid solutions correlates well with the reference concentrations (Supplementary Table S2).

The magnetic properties of CuCr_0.99_Ln_0.01_S_2_ were measured using the Faraday method in the wide temperature range of 80–750 K. The temperature stabilization was controlled using a Delta DTB9696 temperature controller (Delta Electronics, Taipei, Taiwan). The voltage from a quartz torque microbalance was measured using Keysight 34465 A digital voltmeter (Keysight Technologies, Santa-Rosa, CA, USA). The magnetic field strength was varied in the range of 4.8 to 8.6 kOe. The magnetic field strength fluctuations did not exceed 2%. The powder samples (~20 mg) were placed in open quartz ampoules and vacuumed at 0.01 Torr pressure. During the measurements, the samples were held in a helium atmosphere at 5 Torr pressure. The diamagnetic contributions into the magnetic susceptibility value were taken into account using the Pascal scheme. The field dependence of the magnetic susceptibility χ(1/H) was measured in order to take into account the possible presence of the ferromagnetic contribution to the magnetic susceptibility value. The effective magnetic moment as a function of temperature was calculated as follows [21,27]:

(3) $$\mu_{\mathrm{eff}}\left(T\right)=\sqrt{8\chi T}$$

The thermal effects accompanying the ODT phase transition were studied using a DSC-500 differential scanning calorimeter (LLC Specpribor, Samara, Russia). The powder samples (~15 mg) were placed in open aluminum crucibles. The DSC signals were measured in the argon flow (50 mL/min) at a constant heating rate of 10 °C/min.

## 3. Results and Discussion

**Figure 2:**
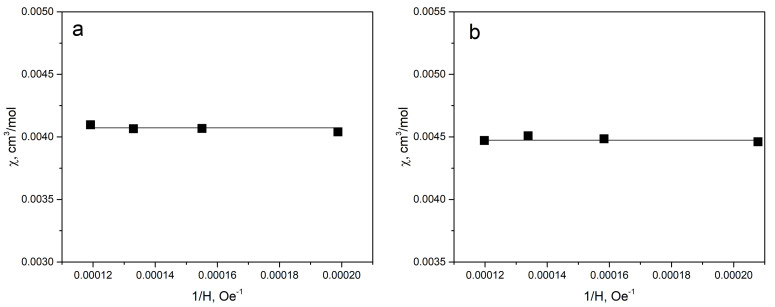
Magnetic susceptibility field dependencies χ(1/H) at T = 300 K: CuCrS_2_ (**a**), CuCr_0.99_Dy_0.01_S_2_(**b**).

Magnetic susceptibility (χ) of chemical compounds is the sum of the different magnetic contributions (paramagnetic, ferromagnetic, and diamagnetic). The diamagnetic contributions are caused by the circulation of the electrons induced by an external magnetic field. The paramagnetic contribution is due to the presence of the unpaired electrons in the material and the field-induced electronic transitions (Van Vleck paramagnetism). The ferromagnetic impurities could significantly affect the magnetic susceptibility value (ferromagnetic contributions). These contributions can be taken into account using the magnetic susceptibility values measured at few different magnetic fields (H). The positive slope of χ(1/H) indicates the ferromagnetic contribution in the measured magnetic susceptibility value. The χ(1/H) behavior of the solid solutions studied were found quite similar. The typical χ(1/H) dependencies are shown in Figure 2. The zero slope of χ(1/H) indicates that the significant ferromagnetic contribution in χ is absent. Thus, one can conclude that the samples studied have no ferromagnetic impurities.

**Figure 3:**
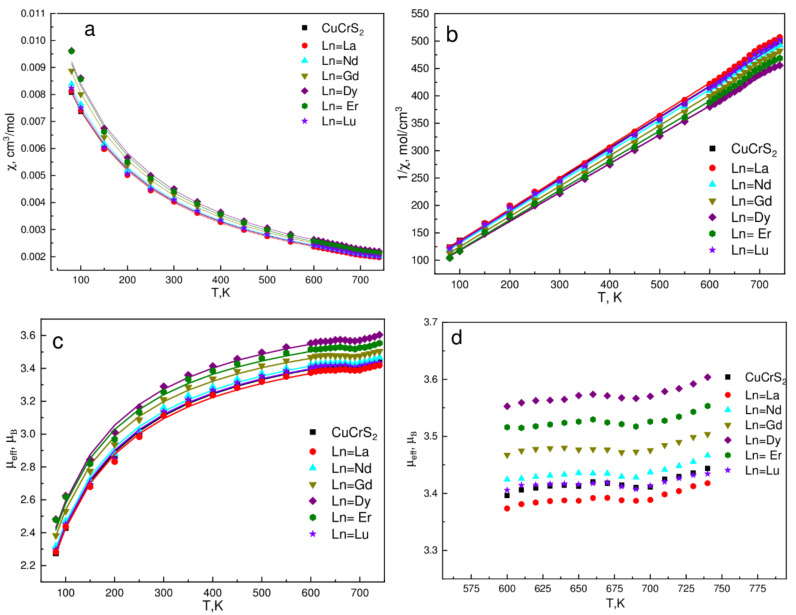
Molar magnetic susceptibility (**a**), inverse magnetic susceptibility (**b**), and effective magnetic moment (**c**,**d**), temperature dependencies. Solid lines are Curie-Weiss law approximation.

The behavior of the measured molar magnetic susceptibility temperature dependencies of CuCr_0.99_Ln_0.01_S_2_ was found quite similar. The typical dependencies of χ(T), 1/χ(T), μ_eff_(T) of CuCrS_2_ and several lanthanide-doped solid solutions CuCr_0.99_Ln_0.01_S_2_ (Ln = La, Nd, Gd, Dy, Er, Lu) are shown in Figure 3. The Curie-Weiss law (Equation (1)) was used to calculate the effective magnetic moment (μ_eff_) and the Weiss constant (θ) values. The μ_eff_ value of 3.77 μ_B_ for CuCrS_2_ was in a good agreement with the experimental data reported previously and corresponded to the theoretical value for Cr^3+^ state (μ_eff_ (Cr^3+^) = 3.87 μ_B_). Note that for CuCrS_2_ experimental μ_ef__f_ value, it lay within the range of 3.75–3.79 μ_B_ [5,20,21,26,27]. The Weiss constant of −140 K correlates well with the reported data and lies within the range of −110 to −150 K [5,20,21,23,27].

**Figure 4:**
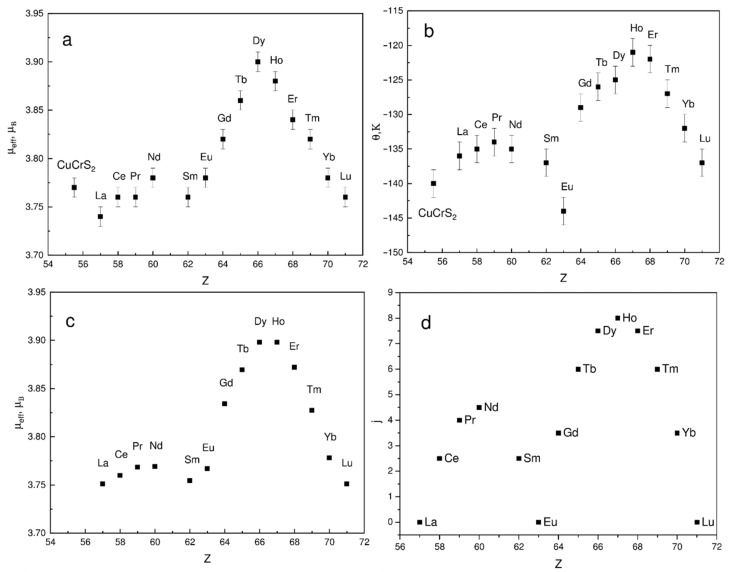
Experimental magnetic moment (**a**), Weiss constant (**b**), theoretical estimation of μ_eff_ (**c**), and the total angular momentum quantum number (**d**) as a function of lanthanide atomic number.

The calculated values of μ_eff_ and θ for the entire series of the samples studied are plotted in Figure 4a,b. The calculated μ_eff_(Z) and θ(Z) have a non-monotonic behavior (Z is the lanthanide atomic number). The maximal μ_eff_ value was observed for CuCr_0.99_Dy_0.01_S_2_, the minimal one for CuCr_0.99_La_0.01_S_2_. Note that in the lanthanide series, the dysprosium and lanthanum atoms have the maximal and the minimal theoretical μ_eff_ value of 10.6 and 0.0 µ_B_, respectively [27,28,29]. Thus, one can conclude that observed μ_eff_(Z) behavior is associated with the lanthanide magnetic moment. Taking into account the isovalent cationic substitution character in CuCr_0.99_Ln_0.01_S_2_ (Ln = La, Ce) solid solutions [8,30], the theoretical μ_eff_(Z) could be calculated (Figure 4c). The theoretical μ_eff_(Z) for CuCr_0.99_Ln_0.01_S_2_ solid solutions were calculated using table μ_eff_ values of Ln^3+^ ions and experimental μ_eff_ value of CuCrS_2_-matrix [27,29]. The obtained experimental μ_eff_(Z) correlates well with the theoretical data calculated using non-interacting spin approximation (Figure 3a,c).

The Weiss constant is related to the exchange interaction between paramagnetic centers. In contrast to CuCrS_2_-matrix, the magnetic properties of CuCr_0.99_Ln_0.01_S_2_ are determined not only by the magnetic Cr^3+^ ions, but also by Ln^3+^ lanthanide ions contributions. Thus, the observed non-linear behavior of θ as a function of Z is determined by the lanthanide contribution. In terms of molecular field theory, the Weiss constant is proportional to the spin (for 3d-metals) or to the total angular momentum quantum number j (for lanthanides). Hence, the theoretical j(Z) dependence for lanthanide ions was plotted in Figure 4d. Note that both j(Z) and θ(Z) dependencies have a similar behavior (Figure 4b,d). The minimal θ values are attributed to the solid solutions doped with lanthanum, europium, and lutetium. This is due to the fact that 4f-orbital is either empty (La) or completely filled (Lu), where j = 0. The observed Weiss constant decrease for CuCr_0.99_Eu_0.01_S_2_ solid solution is due to the spin and orbital magnetic moments compensation (spin-orbit compensation) [31]. Thus, the zero value of j leads to the absence of the contribution of the lanthanide atoms to the exchange interaction integrals in Equation (2). The decrease of the θ absolute value indicates the weakening of the antiferromagnetic interactions after the cationic substitution of chromium with lanthanide atoms with j ≠ 0.

Thus, the behavior of the magnetic properties of the lanthanide-doped solid solutions CuCr_0.99_Ln_0.01_S_2_ (Ln = La…Lu) is majorly determined by the lanthanide ion magnetic properties. Thereby, the most significant effect was observed for lanthanides with the highest j (from Tb to Er).

**Figure 5:**
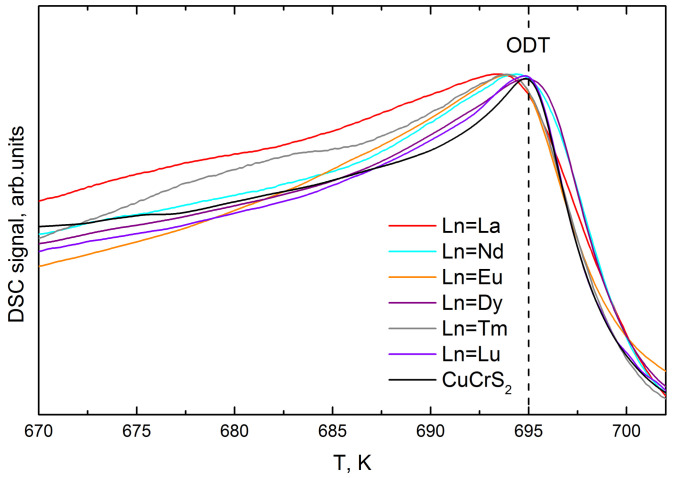
DSC-signal of initial CuCrS_2_-matrix and CuCr_0.99_Ln_0.01_S_2_ (Ln = La, Nd, Eu, Dy, Tm, Lu) solid solutions in the temperature region of the ODT.

As was discussed above, the magnetic properties of CuCr_1-x_Ln_x_S_2_ in the ODT temperature region are of special interest. The magnetic susceptibility of the initial copper-chromium disulfide and CuCrS_2_-based solid solutions in the temperature region of 100 to 800 K is commonly described in terms of the Curie-Weiss law [2,6,20]. However, the improving of the temperature resolution from ~50 K to 10 K allows one to observe the deviation from the Curie–Weiss law in the ODT temperature region (Figure 3a). The corresponding effect could be clearly observed in the effective magnetic moment temperature dependencies μ_eff_(T) (Figure 3c). For clarity, the enlarged curves of μ_eff_(T) within the temperature range ~90 K below and ~50 K above the ODT (T_ODT_~695 K) are plotted in Figure 3d. The corresponding temperature region exhibits an inflection feature. This fact allows one to conclude that the μ_eff_ value is affected by the ODT. As it was reported previously, the ODT in CuCrS_2_ and similar layered dichalcogenides could be studied using DSC technique [3,13,32]. In this regard, the DSC signals of CuCr_0.99_Ln_0.01_S_2_ were measured in the same temperature region (Figure 5). The measured DSC signals exhibit a single peak at ~694–695 K. Thus, the position of the inflection feature on μ_eff_(T) dependencies and peak on the DSC curves are correlated well. At room temperature, copper atoms are localized at the “ordered” tetrahedral sites between CrS_2_-layers, whereas the “disordered” octahedral sites remain unoccupied [10,26,33,34]. With the temperature increase, the occupation probability of octahedral sites increases. Hence, in the temperature region above the ODT, copper atoms are statistically distributed between tetrahedral and octahedral sites. The “ordered” tetrahedral sites were shifted to CrS_2_-layer, whereas octahedral sites were centered between two adjacent CrS_2_-layers. Since μ_eff_(T) decreases at T < T_ODT_, one can conclude that copper atoms in octahedral sites provide more efficient channels for the indirect exchange interaction than atoms in tetrahedral sites. The exchange interaction increase resulted in an increase in the Weiss constant (see Equation (2)). The further temperature increase lead to μ_eff_(T) an increase at T > T_ODT_ due to the magnetic moments disordering of Cr and Ln ions. Thus, the observed μ_eff_(T) behavior could be associated with the redistribution of the copper atoms over different crystallographic sites caused by the ODT.

## 4. Conclusions

A comprehensive study of CuCr_0.99_Ln_0.01_S_2_ (Ln = La…Lu) magnetic properties in a wide temperature range of 80–740 K was carried out. It was established that the effective magnetic moment and the Weiss constant of the lanthanide-doped solid solutions are significantly affected by the total angular momentum quantum number *j* and the effective magnetic moment of the lanthanide ion. The most significant effect on the magnetic properties was observed for solid solutions doped with Tb, Dy, Ho, and Er. Thus, these lanthanides are the most promising candidates for the modification of CuCrS_2_-based solid solutions magnetic properties. The magnetic properties’ behavior in the immediate vicinity of the ODT was reported for the first time. The magnetic susceptibility and the effective magnetic moment of CuCr_0.99_Ln_0.01_S_2_ were found to decrease at temperatures below the ODT. The observed behavior of the effective magnetic moment temperature dependence in the ODT temperature region was described as a result of copper atoms redistribution over different types of crystallographic sites.

## Data Availability

The data presented in this study are available in Supplementary Materials.

## Supplementary Materials

The following are available online at https://www.mdpi.com/article/10.3390/ma14175101/s1, Figure S1: Powder diffraction patterns of CuCrS_2_-matrix and CuCr_0.99_Ln_0.01_S_2_ solid solutions, Table S1: Lattice parameters of CuCr_0.99_Ln_0.01_S_2_, Table S2: Elemental composition of CuCr_0.99_Ln_0.01_S_2_.
