# Supplementary material for: Magnetic Properties of Novel Layered Disulfides CuCr0.99Ln0.01S2 (Ln = La…Lu)
Source: Materials (Basel). 2021 Sep 6;14(17):5101. doi: 10.3390/ma14175101 (PMC8434257; doi:10.3390/ma14175101)
Supplement: Supplementary file 1 [file materials-14-05101-s001.zip › materials-1365944-supplementary.pdf]

Supplementary

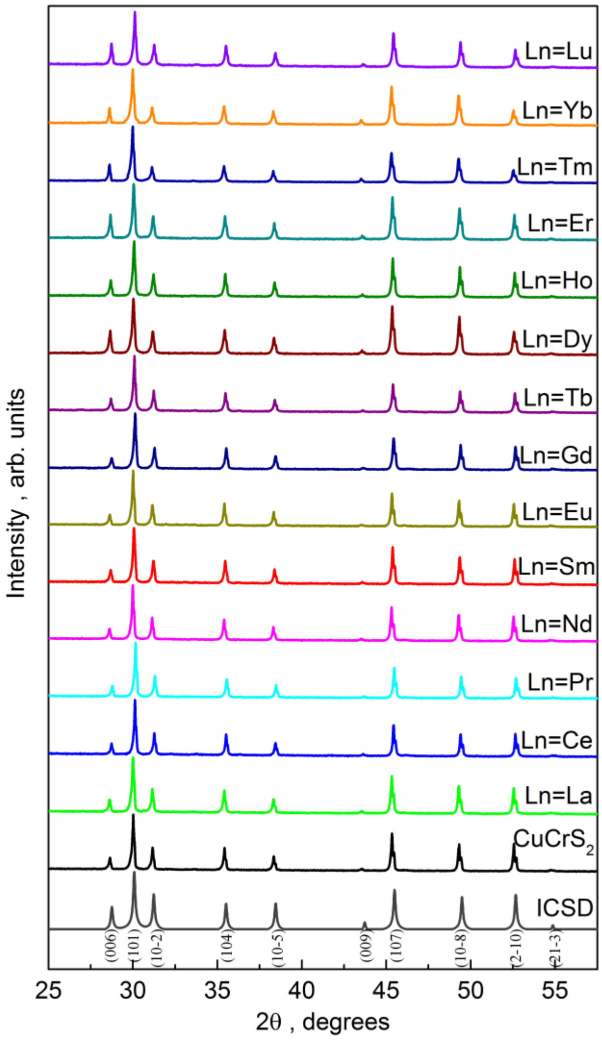

Figure S1. Powder diffraction patterns of $CuCrS_2$-matrix and $CuCr_{0.99}Ln_{0.01}S_2$ solid solutions.

Table S1. Lattice parameters of $CuCr_{0.99}Ln_{0.01}S_2$.

Ln	a, Å	c, Å	Ln	a, Å	c, Å	Ln	a, Å	c, Å
$CuCrS_2$	3.48(3)	18.71(6)	Sm	3.47(9)	18.68(6)	Ho	3.47(7)	18.68(1)
La	3.48(2)	18.70(0)	Eu	3.47(9)	18.68(5)	Er	3.47(8)	18.68(2)
Ce	3.48(0)	18.69(2)	Gd	3.48(0)	18.69(6)	Tm	3.47(8)	18.67(4)
Pr	3.48(2)	18.70(1)	Tb	3.48(0)	18.69(6)	Yb	3.47(0)	18.66(9)
Nd	3.48(0)	18.68(8)	Dy	3.48(0)	18.67(8)	Lu	3.47(8)	18.69(0)

Table S2. Elemental composition of $CuCr_{0.99}Ln_{0.01}S_2$.

	Mean element concentration, mass%				Reference concentration, mass%			
	**Cu**	**Cr**	**Ln**	**S**	**Cu**	**Cr**	**Ln**	**S**
$CuCrS_2$	35.7	29.4	0.0	34.9	35.4	28.9	0.0	35.7
$CuCr_{0.99}La_{0.01}S_2$	35.6	29.9	0.8	33.7	35.2	28.5	0.8	35. 5
$CuCr_{0.99}Ce_{0.01}S_2$	35.1	30.0	0.7	34.2	35.2	28.5	0.8	35.5
$CuCr_{0.99}Pr_{0.01}S_2$	35.1	29.4	0.8	34.7	35.2	28.5	0.8	35.5
$CuCr_{0.99}Nd_{0.01}S_2$	35.1	29.0	0.8	35.0	35.2	28.5	0.8	35.5
$CuCr_{0.99}Sm_{0.01}S_2$	35.7	29.3	0.6	34.5	35.2	28.5	0.8	35.5
$CuCr_{0.99}Eu_{0.01}S_2$	34.4	29.2	0.8	35.4	35.2	28.5	0.8	35.5
$CuCr_{0.99}Gd_{0.01}S_2$	35.5	29.4	1.0	34.3	35.2	28.5	0.9	35.5
$CuCr_{0.99}Tb_{0.01}S_2$	35.3	29.4	0.7	34.7	35.2	28.5	0.9	35.5
$CuCr_{0.99}Dy_{0.01}S_2$	35.4	29.2	0.7	34.7	35.2	28.5	0.9	35.5
$CuCr_{0.99}Ho_{0.01}S_2$	36.1	28.4	0.8	34.7	35.1	28.5	0.9	35.5
$CuCr_{0.99}Er_{0.01}S_2$	35.0	30.0	1.0	35.0	35.1	28.5	0.9	35.5
$CuCr_{0.99}Tm_{0.01}S_2$	35.0	29.4	0.8	34.8	35.1	28.5	0.9	35.5
$CuCr_{0.99}Yb_{0.01}S_2$	34.8	29.1	0.8	34.8	35.1	28.5	1.0	35.5
$CuCr_{0.99}Lu_{0.01}S_2$	35.2	29.5	0.8	34.3	35.1	28.5	1.0	35.5
